# Análisis de la tendencia temporal de la mortalidad por diabetes mellitus en Argentina, 1990-2013

**DOI:** 10.26633/RPSP.2017.73

**Published:** 2017-04-28

**Authors:** Hernán Hernández, Guillermo Macías

**Affiliations:** 1 Universidad Nacional de La Matanza (UNLaM) Universidad Nacional de La Matanza (UNLaM) Buenos Aires Argentina Universidad Nacional de La Matanza (UNLaM), Buenos Aires, Argentina.

**Keywords:** Diabetes mellitus, mortalidad, tendencias, epidemiología descriptiva, Argentina, Diabetes mellitus, mortality, trends, epidemiology, descriptive, Argentina, Diabetes mellitus, mortality, trends, epidemiology, descriptive, Argentina

## Abstract

**Objetivos:**

El objetivo de este trabajo es describir la tendencia temporal de la mortalidad por Diabetes Mellitus (DM) en la Argentina en el período comprendido entre 1990 y 2013, por edad y sexo.

**Métodos:**

Se calcularon las tasas brutas, específicas por edad y ajustadas por edad de mortalidad por DM en la Argentina para el período 1990-2013. Los datos de mortalidad se obtuvieron del Informe Estadístico de Defunción de la Dirección de Estadísticas e Información de Salud. Se realizó un análisis de la tendencia mediante modelos de regresión joinpoint.

**Resultados:**

El análisis de la tendencia de las tasas brutas y ajustadas por edad de mortalidad por DM muestra un modelo estadísticamente significativo en el que se produce un incremento en la mortalidad entre 1990 y 2001, momento a partir del cual puede observarse un descenso. Asimismo, para las tasas ajustadas por edad se encuentra una tendencia significativa de descenso de la mortalidad para mujeres (AAPC: -1,10, IC 95%: -1,70; -0,50). Las tasas de mortalidad específicas por edad se multiplican cada 10 años de edad. Todos los grupos de edad mayores de 50 años muestran una tendencia creciente entre 1990 y 2001.

**Conclusiones:**

La mortalidad por DM afecta principalmente a personas mayores de 50 años y a hombres. Es significativa la tendencia decreciente en las tasas ajustadas de mortalidad por DM para mujeres. Se subraya la importancia de desarrollar políticas de prevención y de detección temprana, como así también la codificación de la muerte por múltiples causas.

## Abstract (en)

**Objectives:**

To describe the time trend of mortality attributable to diabetes mellitus (DM) in Argentina in the years 1990-2013, by age and sex.

**Methods:**

Crude, age-specific, and age-adjusted rates of DM mortality in Argentina were calculated for the period 1990-2013. Mortality data were obtained from the Statistical Report on Deaths issued by the Department of Statistics and Health Information. An analysis of this trend was carried out through joinpoint regression models.

**Results:**

Analysis of the trend of crude and age-adjusted DM mortality rates yielded a statistically significant model in which mortality increased between 1990 and 2001 and declined thereafter. Furthermore, for age-adjusted rates, there was a significant downward trend of mortality in women (AAPC -1.10, 95%CI -1.70 to -0.50). Agespecific mortality rates multiplied with every 10-year increment in age. All age groups older than 50 years showed a growing mortality trend between 1990 and 2001.

**Conclusions:**

DM mortality mainly affects people over the age of 50 and men. A significant downward trend in age-adjusted DM mortality rates was observed for women. These findings highlight the importance of developing policies for prevention and early detection, as well as of proper coding of multiple causes of death.

## RESUMO

**Objetivos:**

Descrever a tendencia temporal da mortalidade por diabetes mellitus (DM) na Argentina no período entre 1990 e 2013, por idade e sexo.

**Métodos:**

Foram estimados os índices de mortalidade por DM brutos por idade e ajustados por idade na Argentina para o período 1990-2013. Os dados de mortalidade foram obtidos do Relatório estatístico de óbitos elaborado pelo Núcleo de Estatísticas e Informado em Saúde. Foi realizada uma análise da tendencia com o uso de modelos de regressao por joinpoint.

**Resultados:**

A análise da tendencia dos índices de mortalidade por DM brutos e ajustados por idade indica um modelo estatisticamente significativo com aumento da mortalidade entre 1990 e 2001, ponto a partir do qual é observado um declínio. Com rela^ao aos índices ajustados por idade, verifica-se uma tendencia significativa de declínio da mortalidade no sexo feminino (AAPC: -1,10, IC 95%: -1,70; -0,50). Os índices de mortalidade por idade sao multiplicados a cada 10 anos de idade. Verifica-se uma tendencia crescente em todas as faixas etárias acima de 50 anos entre 1990 e 2001. *Conclusoes:* A mortalidade por DM atinge principalmente indivíduos do sexo masculino e com idade acima de 50 anos. É significativa a tendencia decrescente nos índices de mortalidade por DM ajustados no sexo feminino. Destaca-se a importancia de desenvolver políticas de prevengo e detecto precoce e da codificado dos óbitos por múltiplas causas.

La diabetes mellitus (DM) es una de las enfermedades crónicas no transmisibles (ECNT) cuyos perfiles de mortalidad y morbilidad se han ido modificando a escala mundial en el último siglo. Según datos de la Organización Mundial de la Salud (OMS), en 2004 la mortalidad por DM ocupaba el doceavo lugar en el mundo y causaba 1,2% de todas las muertes (1). Para 2030, la OMS proyecta que la DM causará 3,3% de todas las muertes y pasará a ocupar el séptimo lugar (1).

La Federación Internacional de Diabetes (IDF) sostiene que el número de personas con DM en el mundo en 2011 ascendía a 366 millones y estima que en 2030 serán 552 millones 2030 (2). En la Región de Las Américas, en 2008 la DM fue la causa de 6% de todas las muertes, precedida por la enfermedad cardiovascular (8%) y la enfermedad isquémica del corazón (9%) (3).

Según las estadísticas vitales de la Di-rección de Estadísticas e Información de Salud (DEIS) del Ministerio de Salud de la Nación Argentina (4), en este país la DM en 2013 ocupó la sexta posición como causa de muerte en las personas del grupo de edad de 55 a 64 años de ambos sexos y representó 3,9% de todas las causas, con una tasa de mortalidad de 39,5 por 100 000 habitantes.

Para responder al incremento de la pre-valencia de las ECNT, en septiembre de 2011 la Asamblea de las Naciones Unidas aprobó la “Declaración política de la reunión de alto nivel de la asamblea general sobre la prevención y el control de las enfermedades no transmisibles”, en la cual se abordan principalmente las enfermedades cardiovasculares, el cáncer, las enfermedades pulmonares crónicas y la diabetes (5).

En esta línea, el Ministerio de Salud de Argentina aprobó la “Estrategia nacional de prevención y control de enfermedades crónicas no transmisibles (ECNT)” mediante la resolución ministerial 1083/09 (6).

En una revisión sistemática publicada en 2012 en el European Journal of Preventive Cardiology se analizaron las causas específicas de mortalidad por DM tipo 1 y tipo 2 en países occidentales (7). En la DM tipo 1, las complicaciones agudas representaban entre 29,5 y 32% de las causas de muerte, seguidas por las causadas por las enfermedades cardiovasculares, las cerebrovasculares y las nefropatías. En la DM tipo 2, la enfermedad cardiovascular es la principal causa de muerte (su frecuencia oscila entre 33,2 y 67,9%) y en menor medida las complicaciones agudas, la enfermedad cerebrovascular, las nefropatías y el cáncer (7). El objetivo de este estudio es conocer la tendencia temporal de la mortalidad por DM por edad y sexo en Argentina en el período comprendido entre 1990 y 2013.

## MATERIALES Y MÉTODOS

Los datos de mortalidad por DM como causa básica se obtuvieron del Informe Estadístico de Defunción (IED) elaborado por la Dirección de Estadísticas e In-formación de Salud (DEIS) del Ministerio de Salud de la Nación Argentina.

Para analizar la mortalidad por DM entre 1990 y 2013, se diseñó un estudio observacional y ecológico cuya unidad de análisis fue el país. Para el período 1990 y 1996, se incluyeron en el análisis los códigos de las enfermedades de la CIE-9 250.0 a 250.9 (8) y para el de 1997 a 2013, los códigos CIE-10 E10 a E14 (9, 10). Los cambios en los códigos de ambas revisiones se publicaron en la tabla de equivalencias de la CIE-10 y son de utilidad para estudiar la mortalidad por DM en su conjunto.

En el análisis se excluyeron aquellos casos sin especificación de sexo o edad. Para calcular las tasas brutas de mortalidad por DM se utilizaron las poblaciones estimadas a 30 de junio de cada año según el sexo y la edad publicadas por el Instituto Nacional de Estadísticas y Censos (INDEC) (11, 12). Para ajustar por edad las tasas de mortalidad por DM se utilizó el método directo y se calcula-ron los intervalos de confianza de 95%. La población para ajustar las tasas de mortalidad corresponde a la población censal argentina de 2010 agrupada en décadas de edad (13). La base de datos del IED se procesó con Microsoft Excel 2010 y PASW Statistic 18 2009. Las tasas brutas, las tasas ajustadas por edad y los intervalos de confianza del 95% se calcularon con el software epidat 4.0.

Asimismo, se realizó un análisis de tendencia de la mortalidad por DM mediante una regresión segmentada *(joinpoint)* de las tasas brutas, de las tasas ajustadas por edad y de las tasas específicas por edad para los grupos de edad mayores de 50 años. Los modelos de regresión de *joinpoint* se componen de algunas fases lineales continuas y permiten describir cambios de tendencia en los datos (por ejemplo, tasas de mortalidad), es decir, con ellos se pueden identificar los puntos donde se producen cambios significativos de la pendiente lineal de la tendencia de las tasas en el tiempo. El análisis comienza probando un modelo con cero *joinpoint*(es decir, una línea recta, sin puntos de inflexión) y luego prueba si uno o más puntos de inflexión agregados en el modelo dan resultados estadísticamente significativos mediante la prueba de permutación (14, 15). A partir de cada coeficiente de regresión, se calcula el porcentaje de cambio anual o, en inglés, APC *(annual percentage change)* y el porcentaje promedio de cambio anual (*average annual percent change* o AAPC, en inglés) y los correspondientes intervalos de confianza, junto con su significación estadística frente a la hipótesis nula de ausencia de cambio en la pendiente (*cero joinpoint*) (14, 15).

Se seleccionaron los modelos de k-*joinpoints* que fueron significativos con un error alfa < 0,05 utilizando la prueba de permutación (14). Para calcular el APC, se utilizó el programa Joinpoint Regression, versión 4.2.0.2. de la División de Control de Cáncer y Ciencias de Población del Instituto Nacional del Cáncer de los Estados Unidos de América (16).

Este estudio reúne las características necesarias para prescindir de evaluación por un comité de ética según la Resolución 1480/2011 “Guía para Investigaciones con Seres Humanos" del Ministerio de Salud de la Nación Argentina (17). Los datos provistos por la DEIS están protegidos por la ley de secreto estadístico (ley No. 17622/68) y su decreto reglamentario 3110/70 (18).

**CUADRO 1:** Análisis *(jointpoint*) de la tendencia de las tasas brutas de mortalidad por diabetes mellitus por sexo (por 100 000), Argentina, 1990-2013

Sexo	Período		Tasa bruta de mortalidad			
	Año de inicio	Año de finalización	Año de inicio	Año de finalización	APC^a^	IC95%^b^
Hombres	1990	2001	16,44	25,25	3,8^^^	2,8; 4,8
	2001	2013	25,25	20,18	-2,3^^^	-3,1;-1,4
Mujeres	1990	2001	17,66	22,80	2,4^^^	1,4; 3,5
	2001	2013	22,80	17,81	-2,2^^^	-3,1;-1,3

***Fuente:*** Elaboración propia basada en los resultados del estudio.

a APC: *Annual percentage change*.

b IC95%: Intervalo de confianza de 95%.

^ APC estadísticamente significativo con un error alfa < 0,05.

## RESULTADOS

**FIGURA 1:**
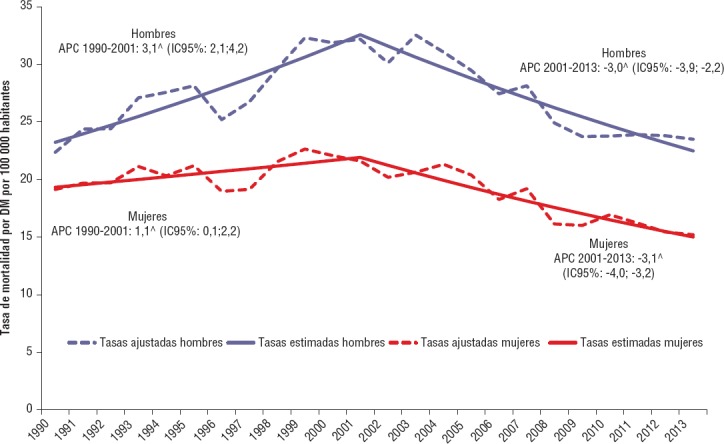
Tendencia de las tasas ajustadas por edad (por 100 000) de la mortalidad por diabetes mellitus por sexo (análisis joinpoint) Argentina, 1990-2013

Las tasas brutas y ajustadas por edad de mortalidad por DM en hombres y mujeres muestran una tendencia creciente entre 1990 y 2001, año a partir del cual se empieza a observar una tendencia decreciente (cuadro 1 y figura 1). El AAPC del período de las tasas brutas de mortalidad por DM para hombres y mujeres es 0,60% (IC95%: 0,00%; 1,20%) y 0,00 (IC95%: -0,70%; 0,60%), respectivamente. Para las tasas de mortalidad por DM ajustadas por edad para hombres y mujeres, el AAPC del período fue -0,10 (IC95%: -0,80%; 0,50%) y -1,10 (IC95%:-1,70%; -0,50%), respectivamente.

Para el período en estudio, 95% de los casos tenían más de 50 años de edad. El análisis de la tendencia de las tasas de mortalidad por DM específicas por edad para hombres y mujeres muestra, para todos los grupos de edad mayores de 50 años, una tendencia creciente entre 1990 y 2001 (aunque para algunos grupos de edad el año de fin del período es el 2000 o 2002), momento a partir del cual comenzaron a descender (cuadro 2 y cuadro 3).

**CUADRO 2:** Análisis *(jointpoint)* de la tendencia de las tasas de mortalidad por diabetes mellitus específicas por edad en hombres (por 100 000), Argentina, 1990-2013

Grupo de edad	Período			
	Año de inicio	Año de finalización	APC^a^	IC95%^b^
	1990	2002	3 18^^^	2,3; 4,1
50-59	2002	2013	-2,84^^^	-3,8;-1,9
60-69	1990	2001	3,57^^^	2,5; 4,6
	2001	2013	-3,43^^^	-4,3; -2,6
70-79	1990	2002	2,99^^^	2,1; 3,9
	2002	2013	-3,60^^^	-4,6; -2,6
80 y más	1990	2000	3,11^^^	1,5; 4,7
	2000	2013	-2,65^^^	-3,7;-1,6

***Fuente:*** Elaboración propia basada en los resultados del estudio.

a APC: *Annual percentage change*.

b IC95%: Intervalo de Confianza del 95%.

^ APC estadísticamente significativo con un error alfa < 0,05.

A su vez, para los hombres de todos los grupos de edad entre 1990 y 2002 el APC fue mayor que el de las mujeres. Los APC para el período 2001-2013 disminuyeron en proporciones más cercanas entre sexos, excepto para el grupo de edad de 80 años y más años en el cual los APC de las mujeres fueron más elevados que en los hombres (cuadro 2 y cuadro 3). A lo largo de todo el período, en los grupos de edad mayores de 50 años, las tasas de mortalidad por DM en los hombres fueron más altas (cuadro 4).

## DISCUSIÓN

El análisis de la tendencia de las tasas de mortalidad brutas y ajustadas por edad por DM, mediante un modelo estadísticamente significativo, indica que, tanto para hombres como para mujeres, entre 1990 y 2001 se produjo un incremento en la mortalidad, momento a partir del cual descendió. Además, en los hombres las tasas de mortalidad por DM brutas y ajustadas por edad fueron mayores a lo largo de todo el período. Las tasas brutas de mortalidad en las mujeres fueron menores que las tasas de mortalidad ajustadas por edad. Estas diferencias son indicios de que la edad es un factor de confusión de la mortalidad y que el incremento de las tasas brutas podría responder a un mayor envejecimiento de este grupo. La esperanza de vida en las mujeres pasó de 75,5 años en 1990-1992 a 78,8 en 2008-2010 en tanto que en los hombres pasó de 68,4 años a 72,08 años en el mismo período (19, 20).

**CUADRO 3:** Análisis (jointpoint) de la tendencia de las tasas de mortalidad por diabetes mellitus específicas por edad en mujeres (por 100 000), Argentina, 1990-2013

Grupo de edad	Período		APC^a^	IC95%^b^
	Año de inicio	Año de finalización		
50-59	1990	2001	1,71^^^	0,3; 3,1
2001	2013	-2,56^^^	-3,7; -1,4
60-69	1990	2001	1,63^^^	0,4; 2,9
	2001	2013	-2,67^^^	-3,7; -1,6
70-79	1990	2002	0,93	-0,2; 2,0
	2002	2013	-3,53^^^	-4,7;-2,3
80 y más	1990	2001	1,03	-0,2; 2,3
	2001	2013	-3,76^^^	-4,8;-2,7

***Fuente:*** Elaboración propia basada en los resultados del estudio.

a APC: *Annual percentage change*.

b IC95%: Intervalo de confianza de 95%.

^ APC estadísticamente significativo con un error alfa < 0,05.

**CUADRO 4:** Tasas de mortalidad por diabetes mellitus específicas por edad y sexo (por 100 000), Argentina, 1990-2013

Grupo de edad	Período					
	1990		2013	
	Hombres	Mujeres	Hombres	Mujeres
50-59	24,33	16,78	25,93	15,69
60-69	71,77	51,98	76,92	50,33
70-79	158,89	127,15	156,33	98,48
80 y más	263,44	281,78	283,07	196,7

***Fuente:*** Elaboración propia basada en los resultados del estudio.

En países como México (21) y Brasil (22, 23, 24) se describen diferencias en la mor-talidad por DM por sexo con una tendencia incremental. Estos estudios muestran que en los últimos años las tasas de mortalidad en los hombres empezaron a ser mayores que en las mujeres (21, 24). En otro estudio sobre mortalidad por DM realizado en Cuba para el periodo 19902002 la tendencia fue decreciente y sin diferencias entre sexos a partir de 2001 (25).

El hecho de que en los hombres las tasas de mortalidad sean mayores puede estar relacionado con la prevalencia diferencial de factores de riesgo asociados con la enfermedad y otras condiciones como el cuidado y el acceso a la salud. En 2005 y 2009 se llevó a cabo en Argentina la Encuesta Nacional de Factores de Riesgo (ENFR) (26, 27) en la población mayor de 18 años. En estas encuestas puede observarse que tanto la prevalencia de DM como de los factores de riesgo asociados es mayor en hombres que en mujeres. En 2005, la prevalencia de DM en hombres fue mayor que en mujeres (12,4 y 11,5%, respectivamente), y en 2009, disminuyó en ambos sexos y su distribución se invirtió, de forma que pasó a ser más alta en mujeres (10,20 y 8,9%, respectivamente).

Los controles de la glucemia (medición de la glucemia alguna vez) eran mayor en mujeres que en hombres, tanto en 2005 (hombres, 62,6% y mujeres, 75,4%) como en 2009 (hombres, 69,1% y mujeres, 81,4%). Por su parte, la prevalencia de obesidad fue más elevada en hombres en ese mismo año (hombres, 15,4% y mujeres, 13,9%) y también en 2009 (hombres, 19,1% y mujeres, 17,1%). Estas relaciones se mantuvieron en la ENFR de 2013 (28). Otros autores que han intentado explicarlo en esta misma línea han atribuido a este perfil de prevalencia de factores de riesgo el hecho de que las tendencias de mortalidad en los hombres hayan tenido mayor crecimiento en los últimos años en comparación con las de las mujeres (24).

Un aspecto relevante y destacable respecto a la mortalidad por DM son las políticas y los programas de atención. A nivel nacional, Argentina cuenta con el Programa Nacional de Prevención y Control de la Diabetes (PRONADIA) (29) y en 2010 la mayor parte de las provincias tenían un programa de DM (30).

A partir de 2002, en Argentina se implementó el Programa REMEDIAR (31) con el cual se distribuyen dos de los medicamentos más utilizados en el tratamiento de la DM tipo 2 (glibenclamida y metformina). Sin embargo, no se distribuye insulina, que tiene una importancia vital en el tratamiento de la DM tipo 1 y potencial en el de la DM tipo 2 (32).

No obstante lo descrito, algunos autores sugieren la escasa cobertura de las políticas de provisión de medicación para la DM. Según Marín y colaboradores (33), el Programa REMEDIAR en 2004 solo proveía tratamiento continuo a 0,60% de los pacientes con DM tipo 2. Una intervención llevada a cabo en Bahía Blanca, en la provincia de Buenos Aires, demuestra que en este municipio, donde la distribución de medicamentos para la DM tipo 2 es gratuita a través de tres niveles de gestión (programa municipal, provincial y nacional), la cobertura pública solo alcanzaba 26,3% de las necesidades considerando la dosis media diaria y 17,5% de las necesidades atendiendo al consumo asociado con la dosis máxima diaria (33).

Además de los problemas de la cobertura de la medicación, en un estudio en que participaron varios países de la Región de Las Américas se observó que la población diabética padece frecuentes complicaciones y que el control metabólico y de los factores de riesgo cardiovasculares asociados es deficiente (32, 34).

En las personas mayores de 50 años, las tasas de mortalidad específicas por edad tanto en hombres como en mujeres se multiplican cada 10 años de edad. Esta fuerte relación entre la mortalidad por DM y la edad ha sido analizada por diversos investigadores (25, 35)

El presente estudio ha analizado la ten-dencia de la mortalidad por DM como causa básica de muerte, tal y como lo publica el IED. Esta manera de aproximarse al estudio de la mortalidad por DM tiene una limitación, ya que la codificación de esta enfermedad, al igual que la del resto de las enfermedades crónicas, encierra cierta complejidad cuando se intenta establecer la cadena causal de la muerte. Estudios realizados en Brasil y en los Estados Unidos de América dan cuenta de este fenómeno (36, 37). Entre otros hechos, en ellos este subregistro se atribuye a que los médicos pueden desconocer que el difunto padecía DM antes de su fallecimiento o considerar que la DM no fue una causa contribuyente de la muerte. Asimismo, sugieren que las personas con DM tienen comorbilidades (hipertensión arterial, dislipemia, enfermedad cardiovascular, etc.) que compiten con la DM en los espacios de los certificados de defunción (36). En un reciente estudio realizado en un distrito de Shangai se describen hechos similares (38). En un estudio sobre historias clínicas de defunciones de pacientes diabéticos en Argentina se comprobó que la DM aparecía mencionada en 21,6% de los certificados de defunción y como causa básica, en 19,8% (39).

Otra limitación del estudio está rela-cionada con la implementación de la CIE-10, que en Argentina data de 1997. Se ha señalado que el cambio en la codificación de la muerte por la implantación de la CIE-10 condujo a un aumento, aunque pequeño, de la mortalidad por DM como causa básica (40-42).

Por último, el período analizado también limita la exploración de la mortalidad de una enfermedad crónica. Sin embargo, la inclusión de años anteriores es difícil porque la cobertura de las estadísticas vitales del país es reducida al igual que su vinculación con otra revisión de la CIE.

La mortalidad por DM muestra una tendencia decreciente de las tasas ajustadas por edad en el período de análisis en hombres y mujeres, aunque dicho descenso solo es significativo en las mujeres. La tendencia de las tasas brutas es creciente en los hombres, pero este aumento no es significativo, y la asociación entre la mortalidad por DM y la edad es muy fuerte.

Sobre la base de estos resultados y en relación con las políticas de salud, y dada la fuerte asociación entre la muerte y la edad, que da cuenta de un aumento en la esperanza de vida de los diabéticos, se recomienda que el sistema de salud desarrolle políticas de prevención y de detección temprana de la DM (fundamentalmente de la tipo 2), para retrasar la aparición de complicaciones y comorbilidades. Respecto a las estadísticas de salud, y considerando lo descrito amplia-mente en la bibliografía sobre la partici pación de DM como causa básica de muerte y como causa interviniente, es aconsejable que el sistema estadístico nacional y los subsistemas implementen la codificación de la mortalidad por múltiples causas, a fin de poder dimensionar el impacto real de la DM en la salud de la población argentina.

## Agradecimiento.

Los autores agradecen muy especialmente a la Dra. Marina Gabriela Zunino la revisión y lectura crítica de este artículo.

## Financiamiento.

Los autores declaran no haber recibido ningún tipo de financiamiento.

## Declaración.

Las opiniones expresadas en este manuscrito son responsabilidad del autor y no reflejan necesariamente los criterios ni la política de la *RPSP/PAJPH* y/o de la OPS.
